# Data on different seed harvesting methods used in grassland restoration on ex-arable land

**DOI:** 10.1016/j.dib.2019.104011

**Published:** 2019-05-24

**Authors:** Ágnes-Júlia Albert, Ondřej Mudrák, Ivana Jongepierová, Karel Fajmon, Ivana Frei, Magdalena Ševčíková, Jitka Klimešová, Jiři Doležal

**Affiliations:** a The Czech Academy of Sciences, Institute of Botany, Department of Functional Ecology, Dukelská 135, 379 82 Třeboň, Czech Republic; b ZO ČSOP Bílé Karpaty, Bartolomějské nám. 47, 698 01 Veselí nad Moravou, Czech Republic; c OSEVA Development and Research Ltd., Hamerská 698, 756 54 Zubří, Czech Republic; d Department of Botany, Faculty of Science, University of South Bohemia, Na Zlaté stoce, 1, 370 05 České Budějovice, Czech Republic

**Keywords:** Bílé Karpaty, Functional traits, Grassland, Old field, Monitoring, Seed addition methods, Seed dispersal

## Abstract

We present data of the grassland restoration experiment performed in the Bílé Karpaty Mts. (White Carpathians, Czech Republic) in dry species-rich meadows. First we harvested seed material in a preserved source meadow (donor site hereafter) by brush harvesting the vegetation once (B1 hereafter), brush harvesting three times during the season (B3 hereafter), and by cutting green hay (GH hereafter). Then we determined the species composition and seed quantity of the harvested material. Furthermore, we transferred the seeds to an experimental site on ex-arable land (receptor site hereafter), and monitored the development of the meadow communities in the following five years. Data are interpreted in: Á-J. Albert, O. Mudrák, I. Jongepierová, K. Fajmon, I. Frei, M. Ševčíková, J. Klimešová, J. Doležal, Grassland restoration on ex-arable land by transfer of brush-harvested propagules and green hay. Agriculture, Ecosystems & Environment 272 (2019), 74–82.

**Table:** Specifications Table

Subject area	*Ecology*
More specific subject area	*Restoration ecology of grasslands*
Type of data	*Tables, figures*
How data was acquired	*Monitoring, survey*
Data format	*Analysed, descriptive*
Experimental factors	*Three methods of seed harvest from species rich grasslands (brush harvesting once a season, brush harvesting three times a season, and green hay transfer) were compared in their efficiency in grassland restoration. Comparison was conducted at the level of harvested seed material, species establishment after the sowing on former arable land, and at the community level.*
Experimental features	*Seeds harvested by devices applicable to large areas of grasslands were determined. Seed material was then tested in a 5 years long sowing experiment to ascertain plant establishment and community development during grassland restoration. Effect of amount of transferred seeds and plant functional traits was evaluated.*
Data source location	*Buffer zone of the Čertoryje National Nature Reserve (48.8478°N, 17.4435°E, 460 m a.s.l.); Malá Vrbka (48.8734°N, 17.4382°E, 300 m a.s.l.); Czech Republic*
Data accessibility	*Data are presented in this article.*
Related research article	*Á-J. Albert, O. Mudrák, I. Jongepierová, K. Fajmon, I. Frei, M. Ševčíková, J. Klimešová, J. Doležal, Grassland restoration on ex-arable land by transfer of brush-harvested propagules and green hay. Agriculture, Ecosystems & Environment 272 (2019), 74–82.**[1]*

**Table:** 

**Value of the Data** • Initiative information for the ecological restoration of species rich grasslands. • Information about the seed harvest from native meadow communities. • Report about the grassland species establishment on former arable land. • Report about the development of plant communities sown on former arable land.

## 1 Data

We compared three different methods of seed harvesting from local meadow communities and assessed their efficiency in meadow restoration on ex-arable land [1]. These methods were: brush harvesting once only, brush harvesting three times during a season, and green hay transfer. We observed the composition of species and functional traits of seed-source meadows, sampled the three harvested seed mixtures and monitored plant communities restored on ex-arable land with this seed over the five following years. Our data represent different outcomes of (a) seed harvest efficiency (Fig. 3, Table 1, Table 2, Table 3), (b) the effects of sown seed mass and functional traits on species establishment (Fig. 4, Table 2, Table 3) and (c) the development of species and functional composition of communities (Fig. 5, Table 4, Table 5).

**Fig. 1:**
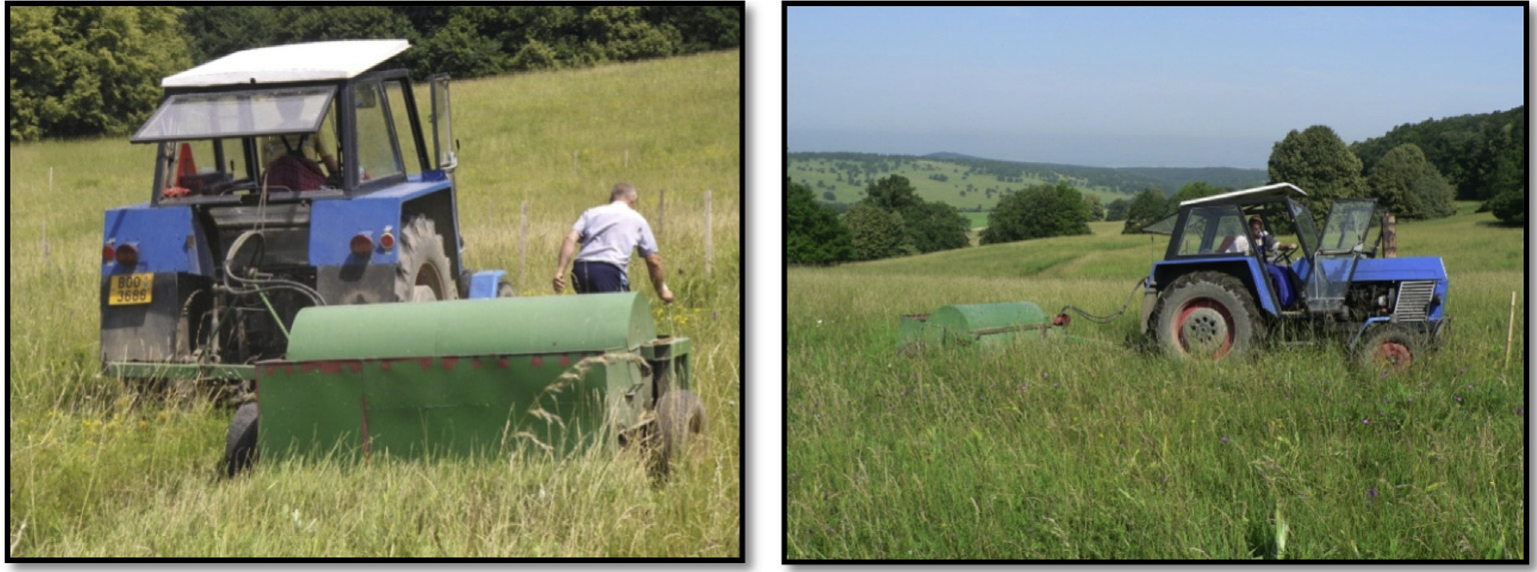
The brush harvester used in our experiment was a special device connected to a commonly used tractor. It harvests 10 m × 25 m seed source plots in approximately 15 minutes.

**Fig. 2:**
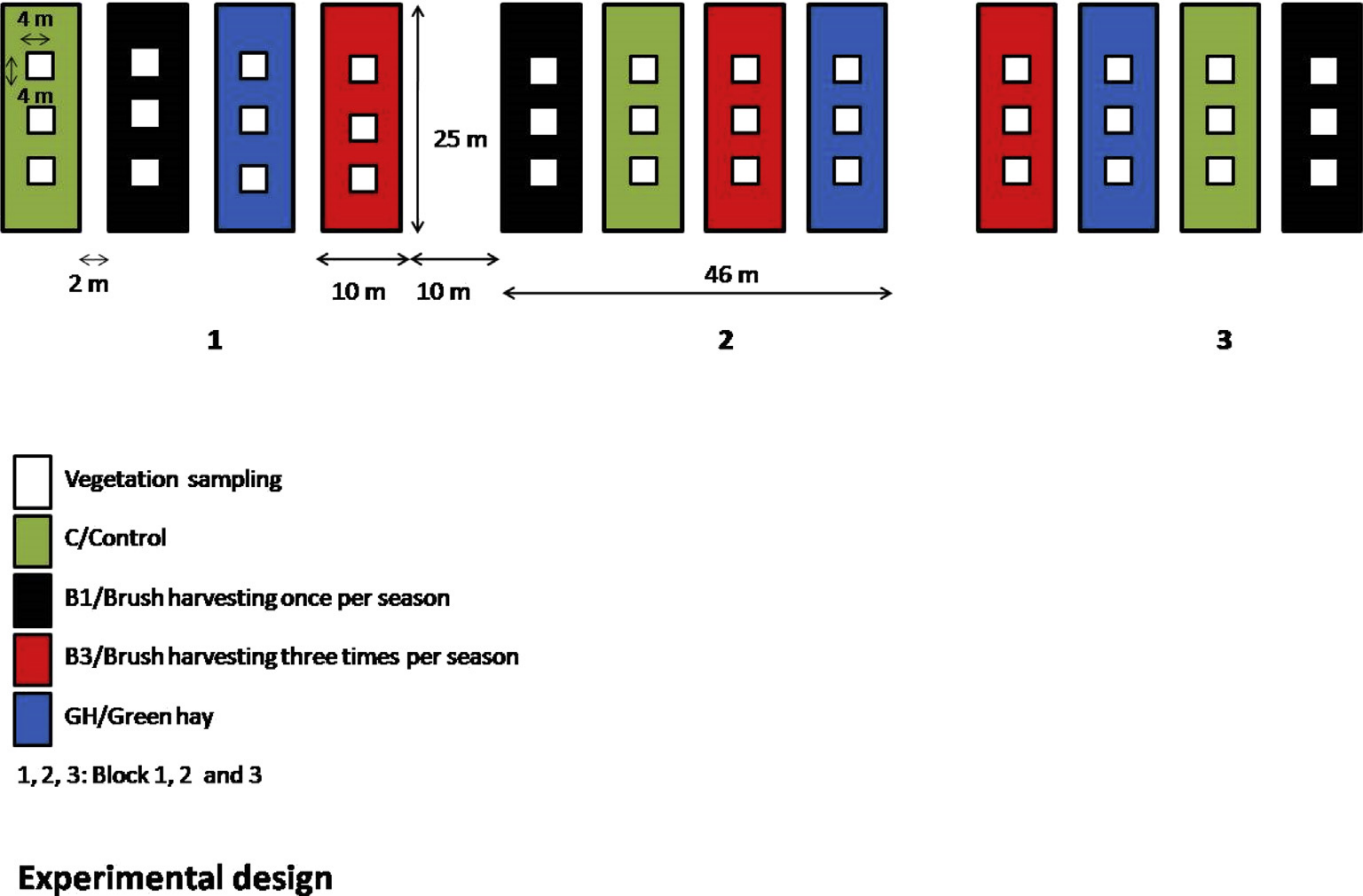
Experimental design of sowing experiment at the receptor site. It consists of three experimental blocks, each containing four 10 m × 25 m plots. Into three plots seed material of the respective method (B1, B3, GH) was applied and one plot was left as unsown control (C). Within each plot three 4 m × 4 m subplots were fixed for vegetation sampling.

**Fig. 3:**
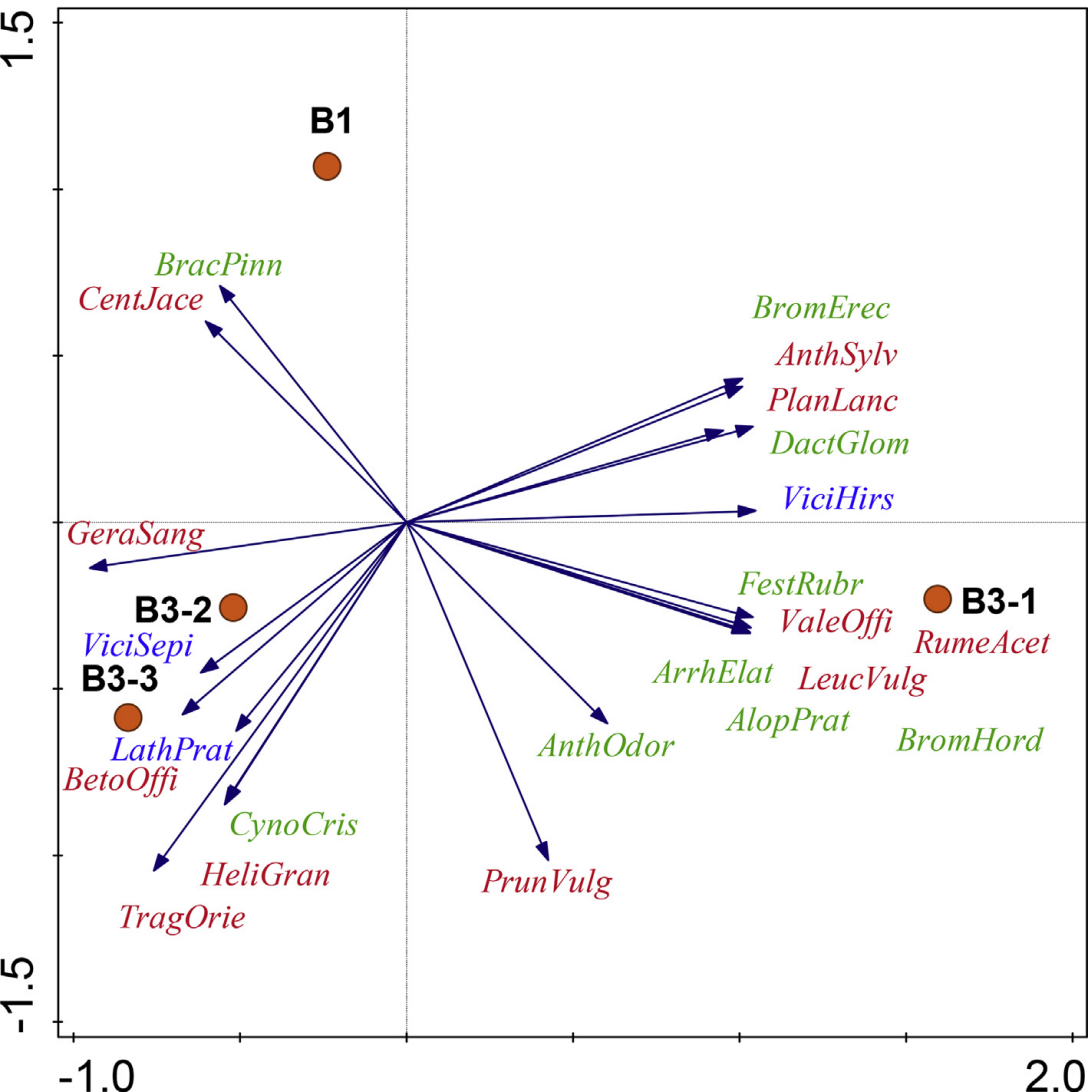
PCA of the seed mass harvested with brush harvesting with particular methods (note that nearly all species had the highest seed mass in the GH method and is therefore not included in this analysis to highlight differences between the other seed harvesting methods). The B3 method is displayed for each harvesting time separately to highlight differences between the harvesting dates. Grasses are marked green, forbs red, and legumes blue. For details, see Methods. Abbreviations of methods: **B1** – seed harvested once, on 27 July 2009, **B3-1** – seed harvest under B3 method on 2 July 2009, **B3-2** – seed harvest under B3 method on 27 July 2009, **B3-3** – seed harvest under B3 method on 21 August 2009. Abbreviations of species: AlopPrat – *Alopecurus pratensis*, AnthOdor – *Anthoxanthum odoratum*, AnthSylv – *Anthriscus sylvestris*, ArrhElat – *Arrhenatherum elatius*, BetoOffi – *Betonica officinalis*, BracPinn – *Brachypodium pinnatum*, BromErec – *Bromus erectus*, BromHord – *Bromus hordeaceus*, CentJace – *Centaurea jacea*, CynoCris – *Cynosurus cristatus*, DactGlom – *Dactylis glomerata*, FestRubr – *Festuca rubra*, GeraSang – *Geranium sanguineum*, HeliGran – *Helianthemum grandiflorum*, LathPrat – *Lathyrus pratensis*, LeucVulg – *Leucanthemum vulgare*, PlanLanc – *Plantago lanceolata*, PrunVulg – *Prunella vulgaris*, RumeAcet – *Rumex acetosa*, TragOrie – *Tragopogon orientalis*, ValeOffi – *Valeriana officinalis* agg., ViciHirs – *Vicia hirsuta*, ViciSepi – *Vicia sepium*.

**Table 1:** Harvested seed material in different seed harvesting methods. **B1** – seed harvested once, on 27 July 2009, **B3** – seed harvest three times during the season (sum of all three harvests), **B3-1** – seed harvested under B3 method on 2 July 2009, **B3-2** – seed harvested under B3 method on 27 July 2009, **B3-3** – seed harvested under B3 method on 21 August 2009, **GH** – green hay transfer on 29 July 2010.

	Method					
	B1	B3	B3-1	B3-2	B3-3	GH
Raw harvested material [g/250 m^2^]	375	1100	–	–	–	200000
Seed [g/250 m^2^]	29	98	82	11	5	2737
Seed of grasses [g/250 m^2^]	7.5	69	64	4	0.1	1209
Seed of forbs [g/250 m^2^]	17.1	21	13	4	4	1139
Seed of legumes [g/250 m^2^]	4.0	8	5	3	1	388
Number of species	26	33	24	26	23	35
Number of grass species	5	9	8	8	4	12
Number of forb species	14	19	13	15	15	20
Number of legume species	7	5	3	3	4	4

**Table 2:** Spearman's rank correlations of mean cover of species transferred with the B1, B3, and GH methods in particular years with seed mass of transferred species sown at restored site (**a**). For all harvesting methods the mean cover of transferred species at the donor site is also correlated with their mass of harvested seeds (**b**). Significant p-values (< 0.05) are marked in bold. **Abbreviations used**: B1 – brush harvesting once, B3 – brush harvesting three times during the season, GH – green hay transfer.

a) correlation of sown species cover at the restored site with their harvested seed mass			
Method	Year	R	P
B1	2010	0.55	0.121
B1	2011	0.22	0.518
B1	2013	0.28	0.374
B1	2014	0.43	0.130
**B3**	**2010**	**0.79**	**<10**^**−3**^
**B3**	**2011**	**0.74**	**<10**^**−3**^
**B3**	**2013**	**0.65**	**0.003**
**B3**	**2014**	**0.58**	**0.006**
**GH**	**2010**	**0.36**	**0.075**
**GH**	**2011**	**0.52**	**0.009**
**GH**	**2013**	**0.55**	**0.008**
**GH**	**2014**	**0.39**	**0.077**

b) correlation of mean species cover at the donor site with their harvested seed mass		
Method	R	P
B1 – donor site	0.06	0.772
**B3 – donor site**	**0.64**	**<10**^**−3**^
**GH – donor site**	**0.47**	**0.007**

**Table 3:** Differences between cover of transferred and non-transferred species tested with the *t*-test verified by a permutation test; (**a**) refers to the differences between the cover of transferred species established at the restored site and the cover of spontaneously establishing ruderal species, (**b**) refers to the cover at the donor site and shows differences between the cover of species the seeds of which were harvested and the species whose seeds were not harvested. Significant p-values (< 0.05) are marked in bold. **Abbreviations used**: B1 – brush harvesting once, B3 – brush harvesting three times during the season, GH – green hay transfer.

a) cover of transferred and unsown species established at restored site				
Method	Year	Transferred species	Unsown species	p
**B1**	**2010**	**0.07±0.02**	**1.0±0.3**	**0.001**
B1	2011	0.4±0.1	0.8±0.2	0.273
B1	2013	1.8±1.0	1.1±0.3	0.949
B1	2014	1.5±0.8	1.1±0.3	0.956
**B3**	**2010**	**0.4±0.2**	**0.9±0.2**	**0.013**
B3	2011	0.8±0.4	1.0±0.4	0.619
B3	2013	1.7±0.8	1.0±0.2	0.891
B3	2014	1.3±0.8	1.2±0.6	0.681
**GH**	**2010**	**0.3±0.1**	**0.8±0.1**	**0.050**
GH	2011	0.8±0.3	0.8±0.2	0.811
GH	2013	1.9±0.8	0.8±0.2	0.301
GH	2014	2.2±1.0	0.6±0.1	0.292

b) cover of transferred and non-transferred species growing at the donor site			
Method	Transferred species	Non-transferred species	p
**B1**	**2.9±0.9**	**0.9±0.2**	**0.003**
B3	2.0±0.7	0.9±0.2	0.069
**GH**	**2.4±0.9**	**0.7±0.1**	**0.004**

**Fig. 4:**
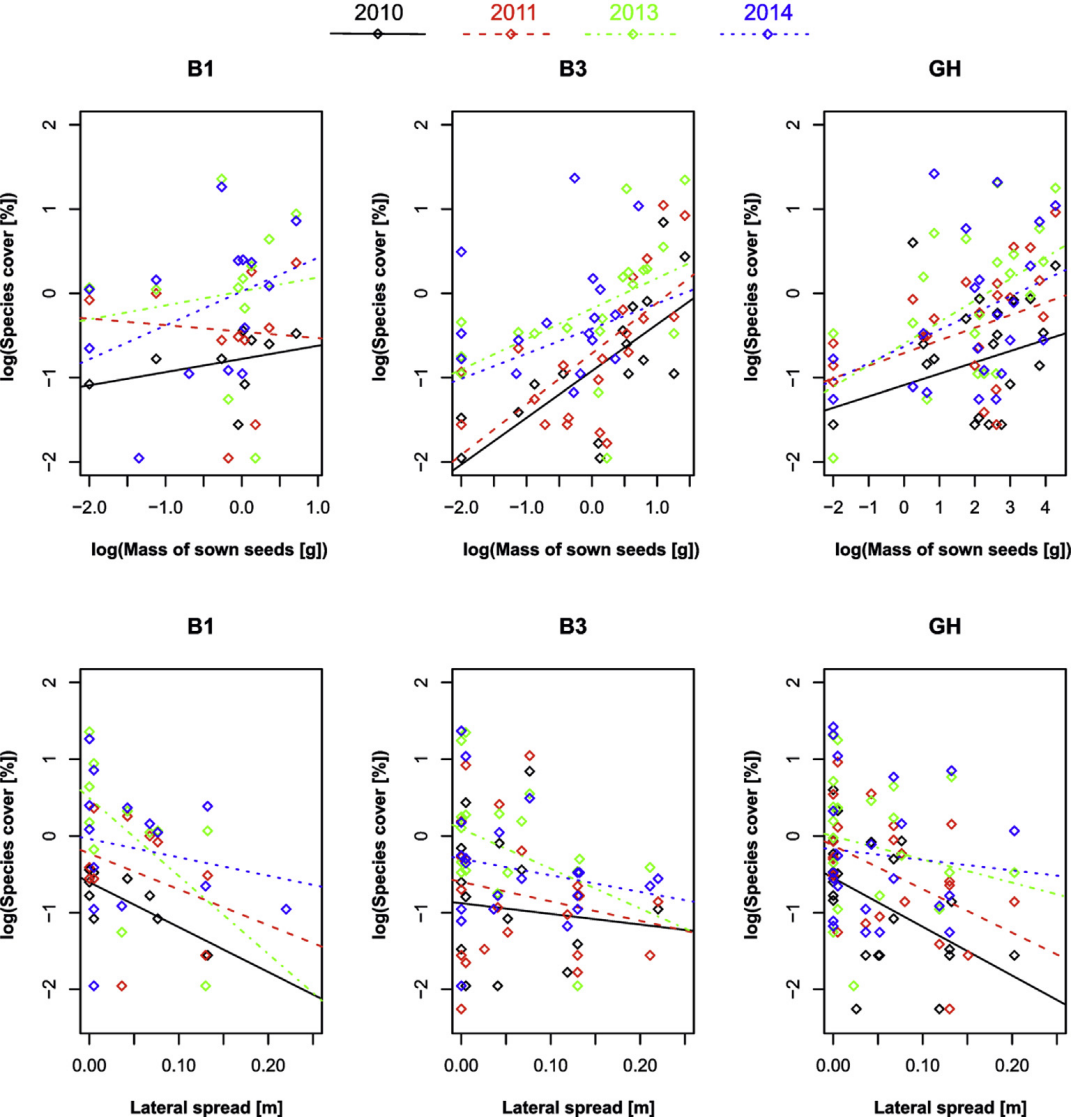
Species cover in particular years correlated to sown seed mass of the species (first row) and lateral spread (second row). All variables (harvesting method, sown seed mass, lateral spread and year) were found to have significant effect on species cover as indicated by LMM (p = 0.014; p < 10^−3^, p < 10^−3^, p = 0.046, respectively; no interaction was significant). **Abbreviations used**: B1 – brush harvesting once; B3 – brush harvesting three times during the season; GH – green hay transfer.

**Fig. 5:**
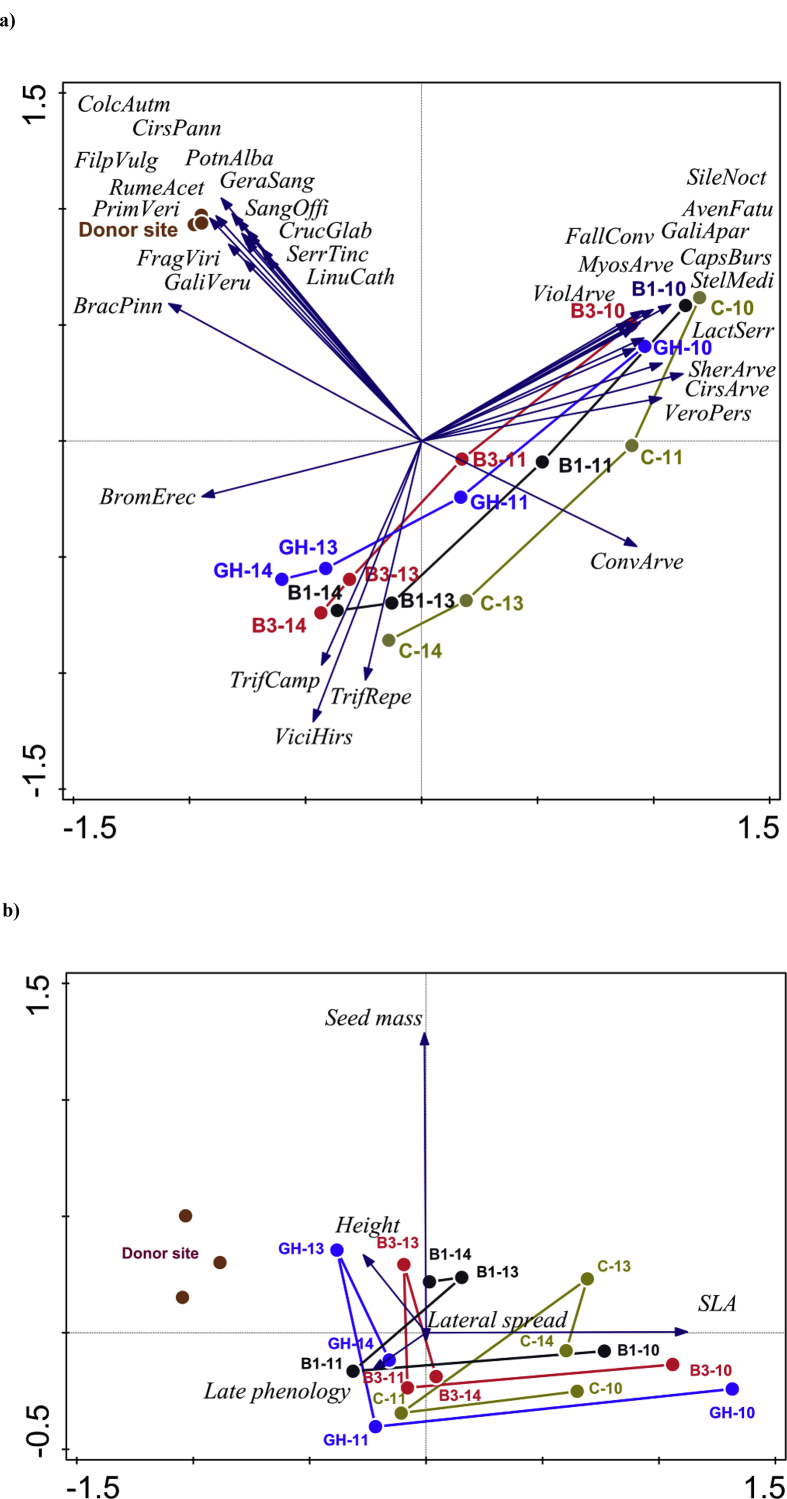
PCA of species composition of plant communities (a) and functional trait composition expressed as CWM (b). Symbols represent the mean position of plots within restoration method and year. Focus of scaling is symmetric. Only species corresponding best with the model are shown. **Abbreviations used**: Donor site – reference seed source meadow, GH – green hay transfer, B1 – brush harvesting once, B3 – brush harvesting three times during the season, C – unsown control, number after the dash indicates sampling year: 10 = 2010, 11 = 2011, 13 = 2013, 14 = 2014, AvenFatu – *Avena fatua*, BracPinn – *Brachypodium pinnatum*, BromErec – *Bromus erectus*, CapsBurs – *Capsella bursa-pastoris*, CirsArve – *Cirsium arvense*, CirsPann – *Cirsium pannonicum*, ColcAutm – *Colchicum autumnale*, ConvArve – *Convolvulus arvensis*, CrucGlab – *Cruciata glabra*, FallConv – *Fallopia convolvulus*, FilpVulg – *Filipendula vulgaris*, FragViri – *Fragaria viridis*, GaliApar – *Galium aparine*, GaliVeru – *Galium verum*, GeraSang – *Geranium sanguineum*, LactSerr – *Lactuca serriola*, LinuCath – *Linum catharticum*, MyosArve – *Myosotis arvensis*, PotnAlba – *Potentilla alba*, PrimVeri – *Primula veris*, RumeAcet – *Rumex acetosa*, SangOffi – *Sanguisorba officinalis*, SerrTinc – *Serratula tinctoria*, SherArve – *Sherardia arvensis*, SileNoct – *Silene noctiflora*, StelMedi – *Stellaria media*, TrifCamp – *Trifolium campestre*, TrifRepe – *Trifolium repens*, VeroPers – *Veronica persica*, ViciHirs – *Vicia hirsuta*, ViolArve – *Viola arvensis*.

**Table 4:** Results of RDA testing species composition (a) and functional trait (CWM) composition (b) of vegetation at the restored site. A summary of three tests is shown for each set of dependent variables.

a)				
Explanatory variables	Covariables	Explained variability	F	P
Method	Year, Block	18.2%	10.0	0.001
Year	Method, Block	49.4%	43.9	0.001
Method * Year	Method, Year, Block	13.6%	2.2	0.001

b)				
Explanatory variables	Covariables	Explained variability	F	P
Method	Year, Block	9.6%	4.8	0.002
Year	Method, Block	19.0%	10.6	0.001
Method * Year	Method, Year, Block	10.3%	1.6	0.010

**Table 5:** P values of linear mixed effect models (LMM) verified by Markov Chain Monte Carlo permutation tests for the main effects of restoration method, year and their interaction. Results of analyses of number of species and cover of individual groups are shown (see Fig. 4, Fig. 5). Significant p values (< 0.05) are marked in bold.

Group of species	Number of species					Cover of species				
	Method	Year	Method × Year	Donor	Donor × Method	Method	Year	Method × Year	Donor	Donor × Method
All	**<10**^**−3**^	**<10**^**−3**^	**<10**^**−3**^	**<10**^**−3**^	0.145	0.772	**<10**^**−3**^	0.324	**<10**^**−3**^	0.478
Grasses	**<10**^**−3**^	**<10**^**−3**^	**<10**^**−3**^	**0.020**	**0.007**	**<10**^**−3**^	**<10**^**−3**^	**<10**^**−3**^	**<10**^**−3**^	**<10**^**−3**^
Forbs	**<10**^**−3**^	**<10**^**−3**^	**<10**^**−3**^	**<10**^**−3**^	0.309	**0.005**	**<10**^**−3**^	0.237	**<10**^**−3**^	0.179
Legumes	**<10**^**−3**^	**<10**^**−3**^	**0.006**	**<10**^**−3**^	**0.019**	**<10**^**−3**^	**<10**^**−3**^	**0.044**	**<10**^**−3**^	0.646
Target species	**<10**^**−3**^	**<10**^**−3**^	**<10**^**−3**^	**<10**^**−3**^	**0.059**	**<10**^**−3**^	**<10**^**−3**^	**<10**^**−3**^	**<10**^**−3**^	**0.008**
Ruderal species	**<10**^**−3**^	**<10**^**−3**^	**0.004**	**<10**^**−3**^	0.178	**<10**^**−3**^	**<10**^**−3**^	**0.006**	**0.001**	0.360
Transferred species	**<10**^**−3**^	**<10**^**−3**^	**<10**^**−3**^	**<10**^**−3**^	**<10**^**−3**^	**<10**^**−3**^	**<10**^**−3**^	**<10**^**−3**^	**<10**^**−3**^	**<10**^**−3**^
Unsown species	**<10**^**−3**^	**<10**^**−3**^	**<10**^**−3**^	**<10**^**−3**^	**0.015**	**<10**^**−3**^	**<10**^**−3**^	**0.008**	0.491	**<10**^**−3**^

## 2 Experimental design, materials, and methods

### 2.1 Study area

The restoration experiment was conducted in the Protected Landscape Area and Biosphere Reserve of the White Carpathian Mts. (Czech Republic). The propagules for the experiment were collected at a donor site situated in the buffer zone of Čertoryje National Nature Reserve (48.8478°N, 17.4435°E, 460 m a.s.l.) in the south-western part of the White Carpathians. The donor site hosted a species-rich vegetation of the *Bromion erecti* association. Dominant species were *Bromus erectus*, *Festuca rubra* and *Festuca rupicola*. The seed material was applied to a receptor site, which was a former arable field next to the village of Malá Vrbka, 2 km NE of the donor site (48.8734°N, 17.4382°E, 300 m a.s.l.).

### 2.2 Experimental design

#### 2.2.1 Seed harvest and collection of seed samples

Propagules were harvested with a brush harvester once at the end of July (27 July 2009); with a brush harvester three times during the season: at the beginning of July, end of July, and at the end of August (2 July 2009, 27 July 2009, and 21 Aug 2009) and as green hay at the end of July (28 July 2009). The harvesting dates were selected based on expert knowledge of the authors [5]. The brush harvester used in our experiment was a special device connected to a commonly used tractor. The area of meadow harvested for each restoration method comprised three plots of 250 m^2^ (10 m × 25 m each). Within each plot three 4 m × 4 m subplots were fixed for vegetation sampling (Fig. 1, Fig. 2).

To determine species richness and quantity of seed in B1 and B3, the collected material was first homogenised by sieving it through a 1-cm mesh, and weighed. Then four subsamples of 10–15 g were taken from the homogenised material. For GH first the total volume of green hay was recorded, then homogenised and four samples of 600–800 g were taken. All subsamples represented an amount of seed harvested from an area of 5–10 m^2^. The total weight of harvested green hay was estimated based on weight and volume of these samples. Subsamples were dried on a plastic tarpaulin. Larger plant remnants were removed, and the resulting material was homogenised by sieving it through a 1-cm mesh. Sieved samples of all methods were cleaned with a Retsch DR 100 ventilator, and seeds were extracted. Seeds were sorted by species, and weighed. The seed weight of each species was then recalculated to harvested area of the plot (250 m^2^) based on its proportion in the subsample. Sampling methods and experimental design followed the methodology and international infrastructure of the SALVERE project [11], [12], [13]. Details on harvested seed mass for the three different methods are given in Fig. 3 and Table 1.

#### 2.2.2 Receptor site and application of seed-addition methods

The receptor site was prepared by ploughing and subsequently harrowing in the spring and summer of 2009. Green hay was spread manually over the receptor site immediately after mowing (28 July 2009) as it is not possible to store it. Homogenised seed material from B1 and B3 was sown manually on 1 September 2009, which is the period commonly used for seed sowing in the area. The area from which the seed material was harvested at the donor site equalled the area to which it was applied at the receptor site (see the Results chapter below for harvested and subsequently sown amounts of seed material).

The experimental plots were arranged into three blocks, each containing four plots (10 m × 25 m). Seed material was applied to three plots (with one seed harvesting method per block) and one plot was left as unsown control (**C** hereafter). The distance between plots within a block was 2 m. Blocks were 10 m distant from each other. Within each plot three 4 m × 4 m subplots were fixed (Appendix 2).

#### 2.2.3 Vegetation sampling

On both donor and receptor site, the cover of vascular plant species was visually estimated in subplots (4 m × 4 m; three subplots per plot; Fig. 2). At the donor site the vegetation was sampled in June (time of maximal productivity) 2009, prior to the seed harvesting. At the receptor site, the vegetation was sampled in June 2010, 2011, 2013 and 2014. Nomenclature follows [9].

#### 2.2.4 Plant functional traits and functional groups

To assess the establishment and consequent performance of plant species differing in their life strategies on ex-arable land (receptor site), we classified each species into one of three functional groups: grasses (*Poaceae* family), legumes (nitrogen-fixing *Fabaceae* family) and forbs (non-leguminous and non-woody dicots). To evaluate the establishment of target and undesired species, we also classified species as meadow and ruderal (or weedy) species based on [2] and expert knowledge of the authors [5], [6], [10]. Meadow species were the target species of this restoration project. In addition, we grouped species into transferred species (recorded in the seed material of each method) and unsown species (not recorded in the seed material of a method). In the control, species recorded in the seed material of any other method were considered as transferred species (this was done to enable comparison with other methods).

To test if the establishment and spread of sown species was an ecologically non-random process, we used five functional traits reflecting key plant life strategies. Plant height (middle value of the provided span) and phenology (first month of the flowering season) were obtained from Ref. [9], specific leaf area (SLA) and seed mass were extracted from the LEDA database [7], and lateral spread from the CloPla database [8]. To quantify the functional trait composition of a plant community, we computed the community-weighted means (CWM) of these traits. Traits were weighed by species cover [3].

### 2.3 Data analysis

Harvested seed mass of the species was correlated with their mean cover at the donor site using Spearman's rank correlations (for each restoration method separately). At the donor site, differences between the mean cover (log transformed) of transferred and non-transferred species were compared by a permutation-based *t*-test using the “independence_test” function (for each restoration method separately) in the R package coin (version 1.1–2, CRAN, [4]). The total amount of seeds, number of species and their functional groups harvested with individual restoration methods are summarised in Table 1. Principle component analysis (PCA) was used to reveal species preference to restoration method and seed harvesting time (Fig. 3).

To assess the dependency of establishment and spread of transferred species at the receptor site on their sown seed mass, the mean cover of transferred species in B1, B3 and GH in particular years was correlated with seed mass of transferred species using Spearman's rank correlations. For all harvesting methods also mean cover of transferred species at the donor site was correlated with their mass of harvested seed. Differences in the mean cover (log transformed) of transferred and non-transferred species (at the receptor site for B1, B3 and GH in particular years) was assessed with a permutation-based *t*-test using the “independence_test” function in the R package coin.

To assess which species have the highest potential to establish from the harvested seed mixtures transferred to the plots, the cover of transferred sown species (mean cover per year and restoration method) was analysed by means of LMM. The used restoration method, year of sampling and five functional traits were included as predictors. Species identity was used as a random factor in all tests. The most parsimonious model was selected by backward selection and then the predictors included in the model (together with possible interaction of categorical and continuous variables) were tested.

To assess changes in species composition and functional trait composition (CWM) at the receptor site, we applied Redundancy analyses (RDA), in which year (as categorical predictors) and their interaction were used as explanatory variables. When testing for the effect of restoration method, year was used as a covariable. When testing for the effect of year, method was used as a covariable. When testing for interaction, the main effects of year and restoration method were used as covariables. Block was used as a covariable in all the cases. Cover data were log (x + 1) transformed to reduce the importance of dominant species. The data was standardised by a sample norm and centred by dependent variables (i.e. by species cover or by CWM). In the permutation scheme, each plot (repeatedly recorded) was considered to be an entire plot. Entire plots were freely exchangeable, but there were no permutations on the split plot level (within records of one plot). The inter-annual and method variability in species and functional trait composition was visualised by means of Principal Component Analysis (PCA, similarly as for RDA, centred by species and standardised by a sample norm) [14]. All multivariate analyses were carried out in the Canoco 5 software [15].

Differences in species number and cover of plant communities and functional groups between years and restoration methods were analysed by means of LMM. Block was used as a variable with random effect. The significance of this model was tested using the Markov Chain Monte Carlo permutation. All categories of species cover and number were log or log (x +1) transformed prior to the analysis of data on a multiplicative scale.
